# *In vitro, in vivo *and microscopic analysis of tumor immunotherapy using combination of two antibodies against the same ErbB2 target

**DOI:** 10.1186/2051-1426-2-S3-P188

**Published:** 2014-11-06

**Authors:** Gabor Toth, Arpad Szoor, Laszlo Simon, Janos Szollosi, Gyorgy Vereb

**Affiliations:** 1 University of Debrecen, Debrecen, Hungary

## 

Trastuzumab (Herceptin^®^), a humanized anti-ErbB2 antibody is a specific targeted therapy against ErbB2 positive tumors, with a history of both success and a high rate of therapy resistance. Another humanized antibody, pertuzumab (Perjeta^®^) inhibits ErbB2 heterodimerization. While these antibodies have been developed based on the in vitro direct cellular effect of their mouse parent antibodies, there is the possibility that their in vivo mechanism of action roots rather in antibody dependent cellular cytotoxicity (ADCC) exerted by natural killer (NK) cells. Our goal was to ascertain the extent of contribution of the direct cellular effect of the antibodies and that of the in vivo evoked ADCC to tumor growth inhibition. We generated the F(ab')2 fragments of the antibodies lacking the Fc part, that have the ability to generate direct cellular effects, but lack the ADCC component. Affinity and lack of Fc fragment on F(ab')2-s were tested with immunofluorescence in flow cytometry. In vitro EC50 was assessed with an MTT based assay. The effect on proliferation of in vitro sensitive BT-474 and resistant JIMT-1 cell lines, both ErbB2 positive, was not affected by the removal of the Fc region. Based on the EC50 values determined as single agents on BT-474 cells, isoboles for a range of combination were also measured for both the whole antibodies and the F(ab')2 fragments and no co-operativity was found. Non-radioactive in vitro ADCC assay on JIMT-1cell line was optimized with CD16.176V.NK-92 high affinity natural killer cell line. Intact antibodies mediated in vitro ADCC-based killing, while F(ab')2 fragments did not. Formation of immunological synapses was verified by confocal microscopy. NK-92 cells were able to form synapses upon recognition of whole antibodies by their high affinity FcγRIII, while owed to the lack of Fc region, synapse formation did not occur with F(ab')2-s. For in vivo ADCC study, JIMT-1 cells were inoculated s.c. in severe combined immunodeficiency mice. The whole antibodies were able to inhibit tumor growth, but their F(ab')2 fragments were ineffective. Combination of the whole antibodies showed a great degree of synergism in vivo, which possibly indicates synergism of two antibodies on the same target protein recruiting FcγRIII receptors and a consequentially better binding and activation of NK cells.

